# High-Throughput Segmentation of Tiled Biological Structures using Random-Walk Distance Transforms

**DOI:** 10.1093/icb/icz117

**Published:** 2019-07-08

**Authors:** Daniel Baum, James C Weaver, Igor Zlotnikov, David Knötel, Lara Tomholt, Mason N Dean

**Affiliations:** 1 Department of Visual Data Analysis, Zuse Institute Berlin, Berlin, Germany; 2 Wyss Institute for Biologically Inspired Engineering, Harvard University, Cambridge, MA, USA; 3 B CUBE—Center for Molecular Bioengineering, Technische Universität Dresden, Dresden, Germany; 4 Harvard Graduate School of Design, Harvard University, Cambridge, MA, USA; 5 Max Planck Institute of Colloids and Interfaces, Department of Biomaterials, Research Campus Golm, Potsdam, Germany

## Abstract

Various 3D imaging techniques are routinely used to examine biological materials, the results of which are usually a stack of grayscale images. In order to quantify structural aspects of the biological materials, however, they must first be extracted from the dataset in a process called segmentation. If the individual structures to be extracted are in contact or very close to each other, distance-based segmentation methods utilizing the Euclidean distance transform are commonly employed. Major disadvantages of the Euclidean distance transform, however, are its susceptibility to noise (very common in biological data), which often leads to incorrect segmentations (i.e., poor separation of objects of interest), and its limitation of being only effective for roundish objects. In the present work, we propose an alternative distance transform method, the random-walk distance transform, and demonstrate its effectiveness in high-throughput segmentation of three microCT datasets of biological tilings (i.e., structures composed of a large number of similar repeating units). In contrast to the Euclidean distance transform, the random-walk approach represents the global, rather than the local, geometric character of the objects to be segmented and, thus, is less susceptible to noise. In addition, it is directly applicable to structures with anisotropic shape characteristics. Using three case studies—tessellated cartilage from a stingray, the dermal endoskeleton of a starfish, and the prismatic layer of a bivalve mollusc shell—we provide a typical workflow for the segmentation of tiled structures, describe core image processing concepts that are underused in biological research, and show that for each study system, large amounts of biologically-relevant data can be rapidly segmented, visualized, and analyzed.

## Introduction

A common structural motif in biology involves the use of repeated, self-similar geometric objects or subunits to cover surfaces or construct protective layers. The scales of fish and reptiles are familiar examples, but such structural tilings are ubiquitous in Eukaryotes, found in plants, animals, and fungi, and can be observed at a large range of size scales, from sub-micrometer mineralized plates, to sub-millimeter cellular foams and tessellations, to centimeter-scaled osteoderms (Yang et al. 2012; Jayasankar et al. 2017). *In situ* and three-dimensional investigations of complex biological architectures has been facilitated by the increased accessibility of laboratory and synchrotron microCT (µCT) technologies. This, however, presents a double-edged sword for data analysis: on the one hand, µCT techniques permit rapid and high-resolution visualization of large regions of interest. On the other hand, quantification of complex, multi-component architectures and the morphologies and arrangements of their structural subunits becomes a massive and demanding task, especially when subunits are in close contact or overlapping and, therefore, difficult to isolate from one another.

Here, we present a general visual data processing approach for the high-throughput separation (segmentation) of large numbers of objects of interest in volumetric datasets, such as those from µCT experiments. We build on specific segmentation solutions that have been used for at least two decades to separate objects in close contact in image data (Malpica et al. 1998; Al-Raoush and Alshibli 2006; Oberlaender et al. 2009; Knötel et al. 2017). Despite the advancements in image processing, a great deal of morphology and ultrastructure research still relies on manual segmentation, which is generally very time-consuming and often hampers statistical analysis due to the bottleneck of the manual segmentation process. As such, the aim of the present work is two-fold. First, with regard to specific approaches, we advocate and detail the advantages of the random-walk distance transform in segmentation workflows, a transformation that has not previously been used for 3D segmentation, but which we show is far more generally applicable and more robust than the more commonly used Euclidean distance transform. Second, since these techniques are used more often in computer science fields, where they are discussed in a manner perhaps less accessible for organismal biology researchers, we would like to make readers of this journal aware of the general structure and availability of workflows like ours and encourage them to pursue options to incorporate and use these, either through their collaborations or in the segmentation software of their choice. While the workflow may seem far less direct than manual segmentation, we demonstrate how this technique can result in enormous gains in efficiency, allowing massive datasets to be analyzed in a comparatively short period of time.

## Segmentation workflow

We present our general image processing workflow and, in the process, summarize several core image processing concepts; references are provided that offer a more thorough treatment of individual techniques.

### Assumptions about the data

**Fig. 1:**
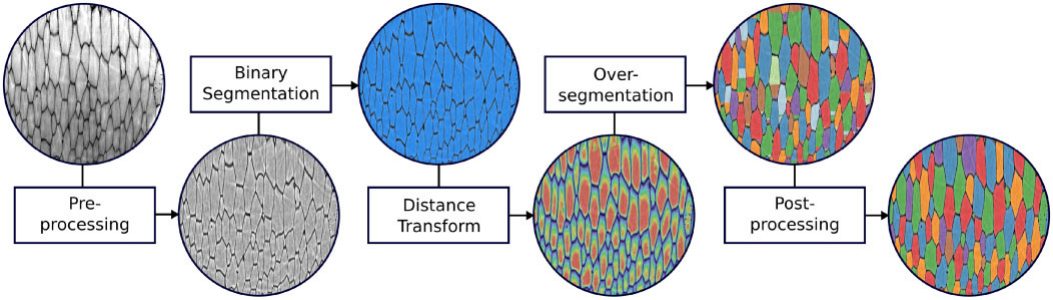
General workflow for the segmentation of tiled biological tissues. Details are explained in the “Segmentation Workflow” section. The images show the processing of the prismatic shell layer of a bivalve mollusc (*Atrina rigida*). It should be noted that, while the depicted images are 2D, the data segmented are 3D (see the online version for color figure).

Our image processing workflow (Fig. 1) is suitable for 3D datasets of repeated structural elements (e.g., tiled structures) exhibiting the following properties:

1. The tiles/structural units are in direct contact. As such, the objects lack well-defined interfaces and cannot easily be separated from one another by considering the intensity values of the image slices alone (e.g., through simple thresholding or more advanced watershed segmentation approaches on intensity values or the derived edges).
2. The material of interest as a whole (i.e., all tiled structures together) can be easily separated from the rest of the image (e.g., background voxels). In our workflow, this step is called binary segmentation (see below).
3. The tiling structures are of similar size. This property is less important than the other two but often makes the processing easier. Here, similar size does not mean equal size, as the size of the objects may still vary by a factor of up to about 10. However, if the structures are of a completely different scale, problems are likely to arise because small structures may be considered as noise and merged into larger ones.

Property 1 above can result from two morphologies, both of which are represented in the case studies of the present work:

- The point of connection between two tiled structures exhibits a constriction (i.e., a narrowing), but lacks a clear structural boundary (see the case studies on stingray tessellated cartilage and starfish ossicles).
- The connection between adjacent tiled structures exhibits a discontinuous boundary that cannot be fully resolved due to either (i) imaging artifacts; (ii) an image resolution that is not high enough to resolve the boundaries; or (iii) a true discontinuous boundary in the biological data (e.g., small connecting bridges between structures, as in the case of the prismatic layer of mollusc shells).

### General workflow

**Fig. 2:**
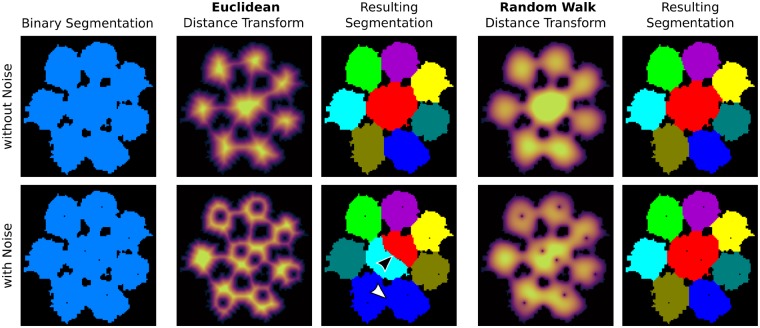
Comparison of Euclidean distance transform and random-walk distance transform showing the susceptibility of the Euclidean distance transform to noise (bottom row). A portion of the tessellated cartilage dataset (see Fig. 3) is used as an example. Top row: distance transforms and resulting segmentations for a binary segmentation without noise. Bottom row: same series as in the top row, but for a binary segmentation with noise (note the black pores inside the foreground objects in the binary segmentation). The segmentation resulting from the Euclidean distance transform contains errors (see arrows) that cannot be overcome by altering the merge threshold. In contrast, the random-walk distance transform is able to produce a correct segmentation even when noise is present and represent the global rather than the local shape of the objects to be segmented (see the online version for color figure).

The general workflow (Fig. 1) can be summarized as follows; more detailed descriptions of each step are presented immediately after. The original image data are rarely in such a good state that one can readily start segmenting the data immediately after the reconstruction step that follows scanning. Instead, a *pre-processing* step involving the application of one or several image filters and normalization algorithms is often required. In our workflow, this pre-processing has the goal to improve the second step of the processing pipeline, the *binary segmentation*. This second step separates the structures of interest (foreground), that is, the tiling structures, from the rest of the image (background). This very important step may strongly influence further processing (see Fig. 2) and, hence, needs to be performed with great care. Yet, it is much less critical using the random-walk distance transform, due to its robustness, in comparison with the Euclidean distance transform (Fig. 2). The third step in the proposed pipeline is the application of a *distance transform*, applied to the foreground voxels of the binary image. The distance transform extracts implicit knowledge about the geometry of the respective objects to be segmented from the binary segmentation. This knowledge is then used in the next step to create an *over-segmentation* of the tiling structures. Here, over-segmentation means that the segmentation is imperfect, with some individual structures of interest represented by several labels instead of just one. Creating an over-segmentation is preferred over an under-segmentation, because merging regions is generally easier than splitting. If additional merging is necessary, it can be performed in the last step, the *post-processing* step.

### Detailed processing pipeline

#### Pre-processing

A vast variety of image filters and normalization algorithms can be applied in this step (Gonzalez and Woods 2008) to reduce artifacts and accentuate natural features to improve segmentation in the following step. These imaging filters are readily available in a variety of common software tools including ImageJ, ITK, and Matlab^®^. The suitable choice of filter depends very much on the particular type of data (e.g., the shape of the tiling subunits or their intensity variation). For the sake of brevity, we list three filters that are commonly used in image processing applications (but perhaps less known in organismal biology work) and that address different problems. If the imaged material is very dense, intensity-value shifts often occur at the image borders, in particular in the corners, so that these regions appear considerably darker or brighter. For this kind of problem, local normalization of each voxel with regard to both the mean and the standard deviation in a certain neighborhood of each voxel is suitable (Sage and Unser 2003), as shown in Fig. 1. One requirement for the normalization to achieve the desired result, however, is to make each neighborhood large enough so that it contains all relevant classes of material/intensity values, ideally in consistent proportion. If the data are noisy, two very popular denoising algorithms are the anisotropic diffusion algorithm (Perona and Malik 1990) and the non-local means algorithm (Buades et al. 2005). The first one smooths the image while preserving edges in the image. The second algorithm removes noise by averaging voxels weighted by the similarity of intensity values in their neighborhoods.

#### Binary segmentation

This step separates the image into foreground and background, where the foreground contains the structures of interest. The simplest binary segmentation algorithm is thresholding, which divides the image voxels into foreground and background according to whether their intensity values are above or below a certain threshold, respectively. Other algorithms include local thresholding for each voxel (Gonzalez and Woods 2008) and the seed-based watershed method (Beucher and Lantuéjoul 1979) which, in addition to the original (filtered/normalized) image or its edge image (an image in which the boundaries of the structures are highlighted), takes some foreground and background voxels as input. More advanced algorithms, sometimes called pixel classifiers, “learn” to identify the foreground through training data. For example, the supervised pixel classifier implemented in the *ilastik* software (Sommer et al. 2011) uses random forests (a supervised machine-learning approach) to segment images into different pixel classes (categories) based on interactive user input. Here, the user marks certain pixels/voxels and assigns them to specific classes (e.g., foreground or background). From this classification and a variety of local image properties, random forests are trained that are then subsequently used to classify all pixels/voxels of the image.

#### Distance transform

Distance transforms take as input a binary image (e.g., the array of foreground pixels/voxels isolated in the previous step) and transform it into a scalar-valued image containing distances measured from each pixel of the foreground to the background. Note the color gradients in the distance-transformed images in Figs. 1 and 2, hotter colors indicating foreground pixels further away from the background. Such transformed image data provide additional information about the objects to be segmented and are sometimes easier to work with in segmentation than the original image data. A good overview of distance transforms can be found in the survey by Jones et al. (2006). The most popular of these transforms is the Euclidean distance transform (Danielsson 1980), for which efficient implementations (Jones et al. 2006) exist, e.g., in ImageJ, ITK, and Matlab^®^. This method measures, for each foreground voxel, the shortest Euclidean distance to any background voxel, hence its name. However, since it measures only a single value, namely the shortest distance, it is very sensitive to noise (Fig. 2). For the same reason, another drawback is that it cannot always represent/detect structural constrictions (i.e., narrow regions) that only occur in one spatial dimension, for example, if the objects are flat. Hence, in this case, the Euclidean distance transform is unsuitable for the purpose of separating tiled objects. For this reason, Knötel et al. (2017) developed a specialized distance map which measures the shortest distance only in the plane that approximates the local orientation of the object at each voxel (e.g., the plane of the surface in a single layer tiling). In order to circumvent the development of such specialized distance transforms, we propose the use of the *random-walk distance transform* (Gorelick et al. 2006), which is far more generally applicable (i.e., allows the determination of all kinds of constrictions) and is also more robust against noise. This transform computes, for each foreground voxel, the average length over all random walks that end in a background voxel. Due to the fact that it considers many distances instead of only a single one, a small amount of noise does not greatly change the appearance of the transform (Fig. 2). As a result, and unlike the Euclidean distance transform, the random-walk distance transform describes the *global* shape rather than the *local* one (Fig. 2). This approach also leads to fewer oversegmented regions following the initial segmentation (compare the segmentations resulting from the Euclidean versus random walk distance transforms in Fig. 2). Despite its favorable properties, to the best of our knowledge, the random-walk distance transform has received almost no attention with regard to object separation in volumetric datasets. We therefore believe the current study is the first to demonstrate its potential as a segmentation tool for µCT datasets.

#### Over-segmentation

In order to separate the individual objects of interest using the distance transformed data generated in the previous step, two methods can be applied: the contour-tree segmentation and the watershed algorithm. The contour-tree segmentation (Van Kreveld et al. 1997; Carr et al. 2003) starts from the local maxima of the distance transform, the voxels furthest away from the background/border. From each local maximum, a region is grown during the contour-tree segmentation by adding voxels to it in decreasing order of intensity values. At some point, neighboring regions that started off from different local maxima come into contact and then can be merged, or not, according to some criteria. The most intuitive method of merging is topological persistence simplification, where the degree of merging is dictated by a single threshold (Edelsbrunner et al. 2000).

An alternative to contour-tree segmentation is the watershed transform (Beucher and Lantuéjoul 1979), which starts from local minima and grows regions by adding voxels in increasing order of intensity values. For this approach, the distance map needs to be inverted, converting the maxima of the distance transform to minima. A common watershed transform that considers recursive merging (i.e., successive merging steps) is called a hierarchical watershed (Beucher 1994; Najman and Schmitt 1996). Regardless of the segmentation tool used, it is important that the user has control over its parameters. In particular, to attain the slightly over-segmented dataset desired in our workflow, the degree of merging must be controllable. It is often unlikely that a single merge threshold can be found that results in a perfect segmentation (i.e., where all objects of interest are neither merged with other objects nor sub-divided into multiple pieces), and therefore post-processing steps are typically necessary.

#### Post-processing

**Fig. 3:**
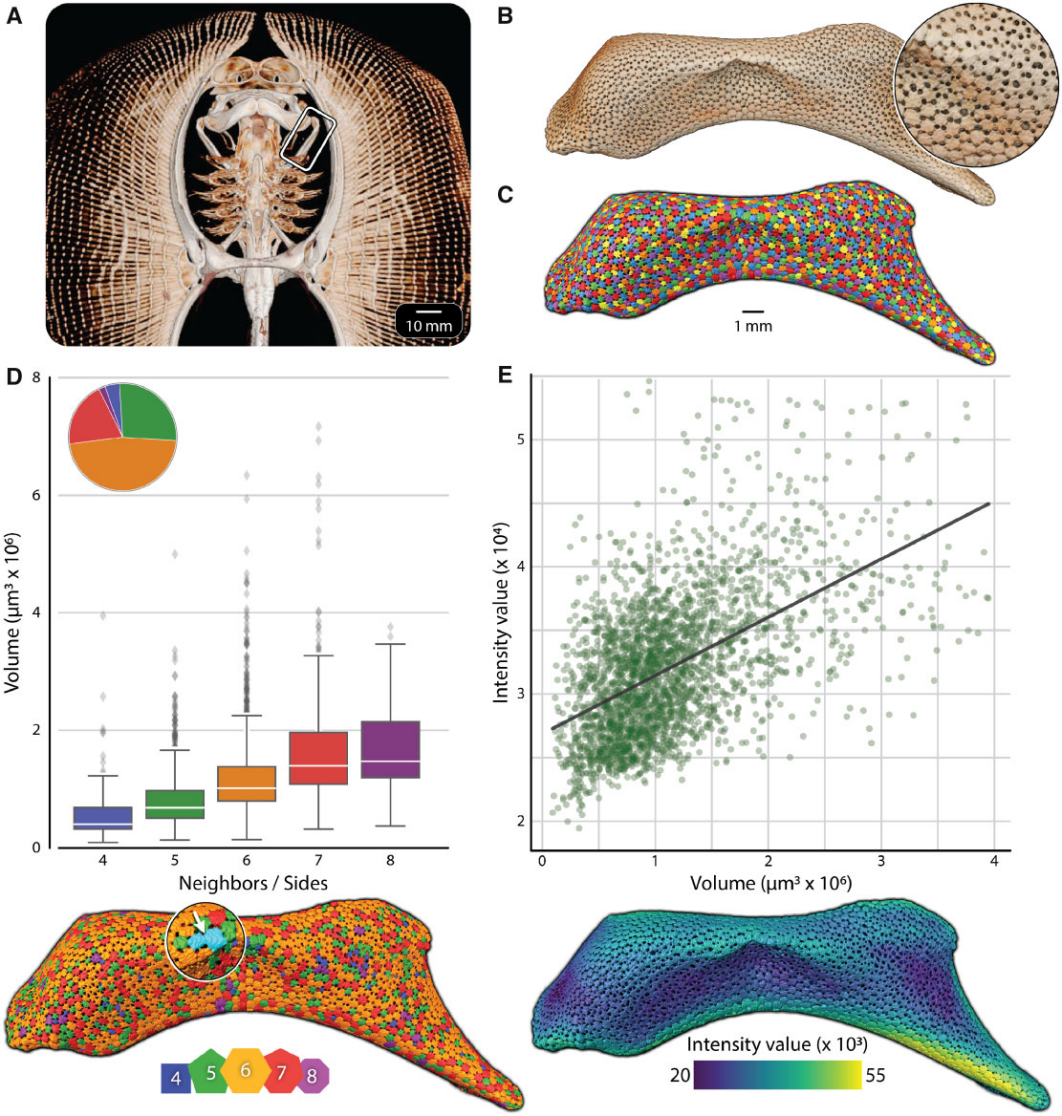
Shape, size, and density quantification of stingray tesserae. (**A**) A microCT scan of the skeleton of a stingray (*Urobatis halleri*) shows the hyomandibula (**B**), the skeletal element investigated. Tesserae covering the surface of the skeleton (B-inset) were segmented (**C**) using the proposed workflow, allowing quantification of all tesserae in a high-throughput fashion in terms of (**D**) tesseral volume, number of neighbors/sides, and (**E**) average intensity value. Tesserae in D and E are color-coded according to scales at the bottom of each image; the pie chart in D shows the proportion of each tile shape in the full dataset. In D, note the under-segmented label containing two connected tesserae, marked by the white arrow in the inset; this segmenting error was easily identified as an outlier in volume and neighbor analyses, allowing targeted repair in manual proofreading (see the online version for color figure).

This step polishes the result of the previous step, starting from the over- (or under-segmented) data in the previous step to generate a more accurate segmentation. This can be accomplished using manual proofreading, where regions are interactively merged or split (e.g., as would be necessary in the Euclidean distance transform segmentation result in Fig. 2), and/or using automatic methods that merge regions depending on criteria other than mathematical persistence (Edelsbrunner et al. 2000). One possibility to automatically merge over-segmented objects of interest is to incorporate model-based information (i.e., prior knowledge of the investigated system) into the watershed algorithm (Lin et al. 2003). For example, for the mollusc shell dataset discussed below, we use prior knowledge of the tissue organization in the post-processing merging step by letting the algorithm preferably merge in the growth direction of the prismatic columns. Another possibility for automatic merging is to formulate the final image segmentation as a mathematical optimization problem that incorporates expert biological knowledge in the form of mathematical constraints (Andres et al. 2011; Beier et al. 2016). In the case studies presented here, we perform little or no post-processing, in order to demonstrate 1) the statistical robustness of the results to normal segmenting errors, which are quite rare when upstream workflow steps are performed carefully; and 2) examples of the types of errors that require post-processing (see Figs. 2 and 3D).

## Case studies

We apply the workflow to three separate µCT datasets: a complete stingray hyomandibula, covered in mineralized tesserae; the entire dermal endoskeleton of a starfish; and the prismatic layer from a portion of a bivalve mollusc shell. Each dataset contains thousands of structural elements, yet it should be stressed that the analyses reported here are for individual specimens and therefore should not be extrapolated to species-level implications. Unless otherwise mentioned, all data processing and volumetric image rendering was performed with an extended version of the Amira software (AmiraZIBEdition 2019.14) (Stalling et al. 2005), in which we implemented the random-walk distance transform. However, many of the segmentation and transformation tools discussed here can also be found in a variety of platforms designed for image analysis. In particular, several image filters, the Euclidean distance transform, and the watershed algorithm are available in platforms such as ImageJ, ITK, and Matlab^®^, as mentioned above. Manual proofreading requires more specialized 3D image segmentation software (e.g., Amira, VGStudio MAX) and the random-walk distance transform is not available in these platforms as standard. It is our hope that the current work will encourage the wider inclusion of this tool in image segmentation platforms.

Processing was performed on an Ubuntu system with two Intel(R) Xeon(R) CPU E5-2650 v2 at 2.60GHz (8 cores each), 64 GB of RAM, and a GeForce GTX 1080 Ti graphics card. For each dataset, we demonstrate the power of high-throughput segmentation through basic downstream morphological analyses, quantifying biologically-relevant aspects of the segmented objects *en masse*.

### Shark and ray tessellated cartilage

#### Background

The skeletons of all living sharks and rays are characterized by a distinct surface tiling (Dean et al. 2009; Seidel et al. 2016; Jayasankar et al. 2017; Knötel et al. 2017; Fig. 3A, B). Unlike most other fishes, which have skeletons composed of bone, shark and ray skeletons are cartilaginous, comprised of a cartilage similar to human hyaline cartilage. Sandwiched between the cartilage and the outer fibrous wrapping of the skeleton (the perichondrium), however, is a layer of mineralized tiles called tesserae. Tesserae are typically hundreds of micrometers wide and thick and cover the surface of nearly the entire skeleton (excepting the centra of the vertebrae), meaning that even a small skeletal element such as the stingray hyomandibula in Fig. 3B (∼1.5 cm long) is armored by several thousand tesserae (Knötel et al. 2017). Although tesserae have characterized shark and ray cartilage for hundreds of millions of years, the patternings and variations of the tessellation have not been quantified due to the challenges of imaging and analyzing the network on a large scale. As a result, for example, although the shape of tesserae have been visualized in a variety of techniques, from camera lucida drawings to light microscopy to µCT images (see references in Dean et al. [2009] and Seidel et al. [2016]), there are still no quantifications of the shape variation of tesserae over size scales relevant to understanding the role tesserae play in the growth and mechanics of the skeleton.

#### Scanning method

For the current work, we scanned a single hyomandibula from a Haller’s stingray (*Urobatis halleri*, sub-adult male, 11 cm disc width) with a Skyscan 1172 desktop µCT scanner (Bruker μCT, Kontich, Belgium), with scanning parameters as described in previous studies (Seidel et al. 2016; Knötel et al. 2017). The resultant reconstructed voxel size was 9.78 μm after resampling.

#### Segmentation method

Pre-processing and binary segmentation was performed as described in Knötel et al. (2017). On the binary segmentation, the random-walk distance transform was computed and the segmentation was carried out using contour-tree segmentation with a persistence threshold of 0.15. No post-processing was done for this dataset. The whole segmentation process took <2 h as opposed to several days that would be needed when doing the processing manually (Knötel et al. 2017).

#### Results and discussion

A vital step in understanding the functional role of tesserae in tessellated cartilage is mapping their arrangements and geometries and determining how they vary (e.g., within skeletal elements or individuals, or among individuals or species). Our current analysis provides the first window into the topological arrangements of tesserae over an entire portion of the skeleton. Our segmentation (Fig. 3C) isolated 2,768 tesserae covering the surface of the hyomandibula, with dimensions (205 ± 42 µm wide×77 ± 24 µm thick, maximum: 453×247 µm) that are in keeping with those previously reported for tesserae of this species (Dean et al. 2009; Seidel et al. 2016).

Our analysis used the number of neighbors surrounding a tessera as a proxy for tesseral geometry (i.e., the number of sides). In this dataset, tesserae had as many as eight sides and as few as four, but six-sided tesserae predominated (1,282 or 47% of segmented tesserae; Fig. 3D). The demonstrated geometries indicate that tesserae tile the surface of this hyomandibula, on average, with a hexagonal packing arrangement, where local “defects” in the tessellation (non-hexagonal tesserae) are balanced (i.e., with five-sided tesserae nearby to seven-sided ones; Fig. 3D). This is similar to the mean hexagonal packings seen in the lattice networks of honeycombs and graphite crystals (Hillert 1965). The distribution of tesseral shapes in this dataset is slightly left-skewed to geometries with fewer sides (i.e., more five- than seven-sided tesserae) resulting in tesserae having an average of 5.84 neighbors/sides. This slight bias toward five-sided tiles indicates that tesserae here follow topological laws for the covering of a “closed” shape by a continuous tiling, where the insertion of five-sided tiles can allow otherwise hexagonal tilings to cover surfaces with positive Gaussian curvature (e.g., as in the mix of pentagons and hexagons on a soccer ball) (Kotschick 2006).

Although there were no obvious trends to the distributions of tesserae of different geometries in this dataset when these were color-coded on the hyomandibula (Fig. 3D), we observed two distinct trends linked to tesseral shape. First, tesserae with more sides were larger, with the volume of eight-sided tesserae on average >3.2× larger than that of four-sided tesserae (Fig. 3D). Second, tesserae with more sides had higher average grayscale intensity values (a proxy for tissue mineral density; Stock 2008), with those of eight-sided tesserae on average 10% higher than those of four-sided tesserae (Fig. 3E). As sharks and rays age, tesserae increase in size by accreting mineral at their margins (Dean et al. 2009; Seidel et al. 2016). Our current analysis suggests that larger (older) tesserae are also those with more sides, indicating that such high-throughput segmentations may be effective for quickly highlighting older regions of the skeleton. We explore these and other growth relationships in an ongoing work, where several datasets are analyzed and compared in tandem.

### Starfish dermal ossicles

#### Background

Echinoderms (starfish and their relatives) all possess an endoskeleton, comprised of mineralized ossicles embedded in the dermis. There is an incredible diversity of ossicle form, connectivity, and organization among taxa. Dermal ossicles can be either loosely distributed as in the sea cucumbers (Holothuroidea), organized into serially repeating units as in the brittle stars (Ophiuroidea) and feather stars (Crinoidea), or fused into a rigid hemispherical shell-like structure as in the sea urchins (Echinoidea) (Brusca et al. 2016). Since ossicle architecture plays a critical role in species identification and for informing evolutionary relationships between echinoderms (Pisera 1979), the ability to reliably depict the spatial relationships of the constituent skeletal elements in the mature skeletal systems is critical.

In the fossil record, echinoderm skeletons are often found disarticulated, represented only by isolated ossicles. While skeletal reconstructions from isolated ossicles can be routinely performed in some groups with only limited prior information (e.g., due to the relatively simple and/or intuitive relationships of the ossicles) (Brower and Veinus 1978), the starfish (asteroidea) represent a notable exception. In starfish, the thousands of individual ossicles are assembled into a complex, loosely articulated network, which encloses the internal organs in a flexible, but protective cage (Blowes et al. 2017). Because of this structural complexity, CT scans of starfish skeletons can be notoriously difficult to interpret, and it is virtually impossible to infer any useful information regarding skeletal organization from the examination of disarticulated starfish skeletal systems. Due to these limitations, the ability to differentially label specific ossicle types in mature starfish skeletal systems is of great value for understanding structure–kinematic relationships in these complex mineralized networks.

#### Scanning method

A 7 cm-diameter (arm tip to arm tip), ethanol-preserved starfish (*Pisaster giganteus*) from the temperate Eastern Pacific was fixed between two low-electron density foam plates and scanned in an XRA-002 X-Tek micro-CT system at 115 keV and 80μA. The resulting transmission image set was reconstructed using CT-Pro software (NikonMetrology) with a final voxel size of 38 μm, and the resulting image stack was exported using VGStudio Max.

#### Segmentation method

**Fig. 4:**
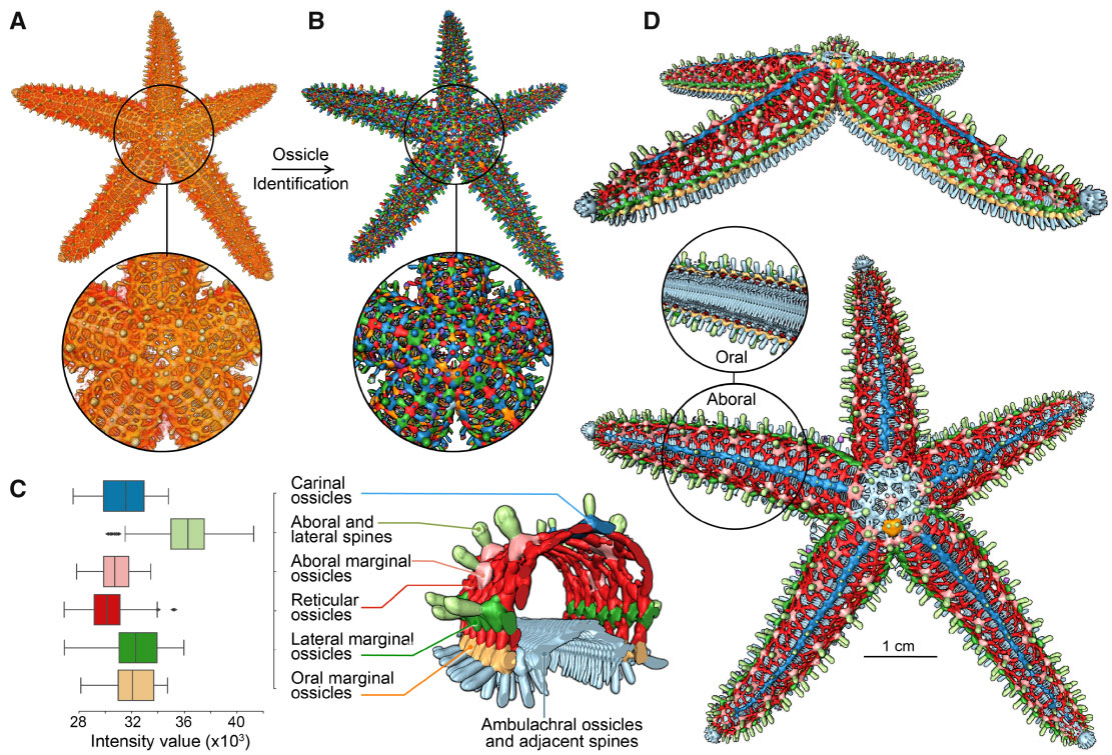
Endoskeleton segmentation and ossicle-type color-coding in starfish. Using µCT data of the entire dermal endoskeleton of *Pisaster giganteus* (**A**), the individual ossicles can be readily identified (**B**). Using morphology-based classification schemes, the different ossicle classes can be segregated and their average electron density profiles (proxy for porosity and mineral density) calculated (**C**). The resulting ossicle groupings can then be color-coded for the entire skeletal system (**D**) (see the online version for color figure).

No pre-processing was performed for this dataset. To hone in on an effective segmentation, the computation of the binary segmentation and the random-walk distance transform were repeated for multiple intensity-value thresholds, ranging from 22,000 to 30,000 in steps of 100. For each threshold, the random-walk distance map was computed and normalized to the range from 0.0 to 1.0. All normalized distance maps were averaged and the result was used as input to the contour-tree segmentation. We used the segmentation where no merging of any kind was performed, and the segmentation was not post-processed in any way. Very small, unconnected components (which corresponded to the pedicellaria) were removed in order to retain only the major skeletal ossicles, resulting in the segmentation depicted in Fig. 4B, which consists of approximately 15,000 segments. The different ossicle types were then manually classified based on shape and position (Fig. 4C).

#### Results and discussion

The computation of a large number of binary segmentations and their respective random-walk distance maps helped identify constrictions (narrow connections) between adjacent ossicles that were only visible for certain intensity-value thresholds. In this way, we were able to first semi-automatically isolate ∼15,000 individual objects, most of which representing complete ossicles of the skeleton, and then classify and color code the ossicles based on their specific morphologies and locations. This proved particularly useful in effectively demonstrating large-scale skeletal symmetry (Fig. 4D), but also allowed rapid quantification and comparison of ossicle features (e.g., grayscale intensity values, Fig. 4C). In addition to providing an intuitive illustration of the distribution of the different ossicle types, this approach also yielded some unexpected results such as the discovery of the kinked and bifurcated organization of the (blue) carinal ossicles in the right arm in Fig. 4D. These results suggest either a developmental anomaly or perhaps evidence of significant damage incurred early in life, and could thus be used as a potential proxy for mapping out the prevalence of injuries in starfish populations. Due to its ability to efficiently clarify network connectivity in asteroid skeletal systems, we are currently adapting this approach for investigating skeletal maturation and growth stages in a single species, and for comparing skeletal articulation patterns in closely or distantly related species.

### Prismatic ultrastructure in mollusc shells

#### Background

Mollusc shells are comprised of highly mineralized tissues, primarily composed of calcium carbonate building blocks, in the form of needles, platelets, or columns, joined together by an organic matrix (Bøggild 1930). Providing the animals with protection against predation, these shells exhibit a unique combination of high strength, high stiffness, and high toughness that is believed to stem from the spatial organization of the different tissue components. As a result, mollusc shells often serve as model systems to study structure–function relationships in biological materials (Currey and Taylor 1974).

**Fig. 5:**
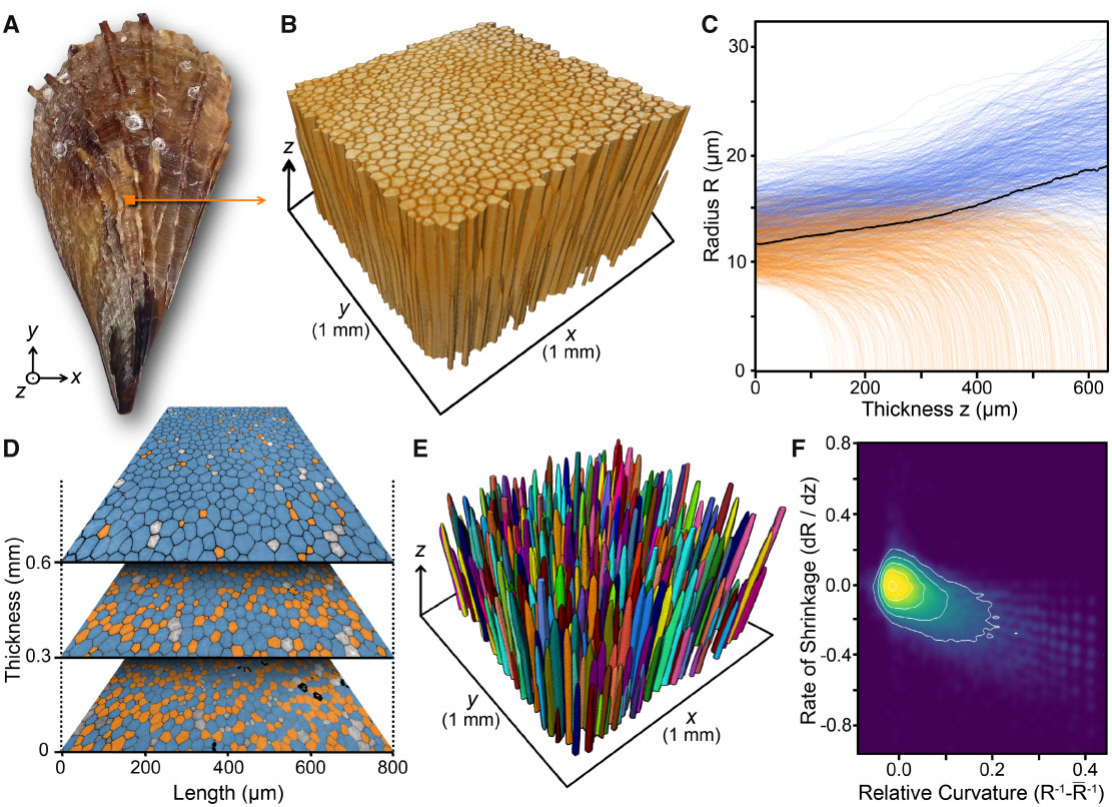
Growth analysis of the prismatic ultrastructure in (**A**) the mineralized shell of the bivalve *Atrina rigida*. (**B**) A 3D reconstruction obtained from the segmented microtomography data of the prismatic ultrastructure. The growth direction of the shell in thickness is denoted by the *z* axis. (**C**) The radius of all segmented prisms as a function of thickness of the prismatic layer, *z*. The black curve represents the average prism radius in the entire prismatic tissue. The radius of each prism was calculated as prism-area-equivalent circles using *R*=√(A/π). (**D**) 2D microtomography sections obtained perpendicular to the growth direction of the prismatic layer at different thicknesses, *z*. (**E**) A 3D reconstruction obtained from the segmented microtomography data of only the shrinking prisms. (**F**) Rate of radius change of the shrinking prisms as a function of their relative curvature. Despite the large data spread, a linear trend is observed. In C and D, blue and orange represent growing and shrinking prisms, respectively (see the online version for color figure).

Typically, shells are made of a number of discrete mineralized ultrastructures, arranged in layers parallel to the outer surface of the shell. Each layer exhibits distinctive morphological characteristics: in the shape and the size of the mineral building blocks, and the thickness of the organic matrix that binds them (Currey and Taylor 1974). For example, some bivalve shells (Fig. 5A) contain an internal nacreous ultrastructure that is composed of mineral platelets (≤1 µm thick) separated by a small amount of organic material (∼40 nm thick) to form a brick-and-mortar-like assembly, and an external prismatic layer (Fig. 5B) that is made of elongated columns joined by a relatively thick (∼1 µm) organic interprismatic matrix (Currey and Taylor 1974; Bayerlein et al. 2014). The three-dimensional distribution of the different components is key to the mechanical performance of both tissue layers and of the entire shell (e.g., Frølich et al. 2017). Whereas 3D morphological studies of nacre are extremely challenging due to the exceedingly small dimensions of the organic phase, the architecture of the prismatic layer is a perfect candidate for X-ray-based microtomography imaging (Bayerlein et al. 2014; Reich et al. 2019).

#### Scanning method

The prismatic layer of the shell of *Atrina rigida* (Nudelman et al. 2007) was studied by synchrotron-based microtomography at beamline ID19 of the European Synchrotron Radiation Facility (ESRF, Grenoble, France) (Fig. 5A and B). The samples were scanned using an X-ray photon energy of 34 keV at a sample detector distance of 91 mm. A total of 5000 radiographic projections were recorded over 180 degrees with an exposure time of 0.1 s. A multilayered monochromator was used to narrow the bandwidth of the radiation impinging on the sample and ESRF in-house code (PyHST2) was used to reconstruct the data. Simple back-projection reconstruction was used for automated data processing. The grain-boundary contrast was enhanced by Paganin-based filtering. Thus, a spatial resolution high enough to resolve the different prismatic mineral units was achieved, with a voxel size of 0.649 µm. To allow for faster processing, the dataset was resampled to a voxel size of 1.298 µm.

#### Segmentation method

The major steps of the processing pipeline for this dataset are depicted in Fig. 1. Pre-processing consisted of application of the Local Normalization ImageJ plugin (Sage and Unser 2003). Binary segmentation was performed using simple thresholding. To compute the over-segmentation, the random-walk distance transform was computed on the binary segmentation, followed by contour-tree segmentation without persistence merging. In the post-processing step, the over-segmented regions were merged into full prisms with a newly developed algorithm that allowed directed merging, preferentially in the direction of the prismatic columns (the growth direction of the prisms); this ensured that, when merging the over-segmented portions of a single prism, neighboring prisms would not be included accidentally. Parameters for this algorithm were set manually based on visual inspection of the results. Details of this algorithm will be presented elsewhere since it is out of the scope of this article. Finally, some remaining, still-broken prismatic columns were manually merged. Post-processing took less than 1 h.

#### Results and discussion

Recent studies of the structural and textural evolution of different biomineralized shell ultrastructures suggest that classical concepts from the field of physics of materials have the capacity to analytically describe the morphogenesis of molluscan shells (Schoeppler et al. 2018; Reich et al. 2019). Focusing on the prismatic architecture in the shells of a variety of organisms, these studies have demonstrated that by following the growth of individual prisms and the average coarsening behavior of the entire prismatic assembly (i.e., the degree to which prisms increase in cross-sectional area as a function of the thickness of the prismatic layer), we can quantify the different thermodynamic boundary conditions that are responsible for the formation of species-specific prismatic morphologies.

Our segmentation of a portion of the prismatic layer of *A. rigida* shell (Fig. 5A) isolated over 1,400 unique prisms, allowing aspects of their morphology to be analyzed in a high-throughput fashion (Fig. 5B–F). The whole segmentation process, including post-processing, required approximately 3 h; by comparison, segmenting 18 prisms manually in a previous study required approximately 1 week (Bayerlein et al. 2014). Measurements taken on the current dataset provide unique information on prism morphogenesis when considered in the scope of grain growth and coarsening theories, which were classically developed to describe the behavior of generic polycrystalline structures during annealing (Atkinson 1988). For example, using a curvature-driven grain growth model, Hillert (1965) argued that the growth of a single grain in a polycrystalline system during coarsening (an increase in average grain size) could be predicted by the average morphology of all grains in the structure. Specifically, he hypothesized that if the size of a specific grain is larger than the average size of all the grains in the material, it will grow, and if its size is smaller than the average, it will shrink and disappear. The prismatic layer of *A. rigida* shells offers an important biological system for testing this prediction, since the change in the morphology of the individual prisms along their length (their growth axis) can be used to understand the growth dynamics of the entire prismatic array over time (Fig. 5C, D). Hillert’s prediction was supported in previous shape analyses of a small number of isolated prisms from the prismatic layer of the shells of other molluscan species (Bayerlein et al. 2014); however, our current data from the prismatic layer of *A. rigida* confirm that this prediction also holds for prisms on a massive, more tissue-relevant scale (i.e., for more than one thousand individual prisms; Fig. 5C). In addition, Hillert proposed a linear relationship between the rate of growth or shrinkage of an individual grain and its curvature. Similarly, our data from all investigated prisms corroborate this prediction for all the segmented shrinking prisms (Figs. 5E and 5F). Further analyses of these data are ongoing, using a variety of analytical correlations for average coarsening behavior, such as the curvature-driven growth model, in conjunction with other geometrical and topological theories, having the capacity to provide fundamental knowledge on the process of molluscan shell biomineralization.

## Conclusion

Our proposed workflow—incorporating a series of common image analysis tools—is capable of segmenting quite different types of structurally-complex, biological datasets with a speed and quality that allows statistical analysis of huge numbers of data points. Our three case studies demonstrate the efficacy of the workflow, while also highlighting several particular features for consideration:

– Our segmentation of stingray tesserae produced results quite similar to those obtained in a previous work (Knötel et al. 2017), where a specific distance transform was deemed necessary to account for particular aspects of tissue morphology. That the random-walk distance transform was effective without specific implementation shows that it can be considered as a very general tool for object separation and therefore can be useful for quite different types of data.
– Our starfish endoskeleton segmentation allowed effective and rapid identification of a huge number of ossicles, yet required manual selection of series of ossicles during the classification process. Ideally, such manual steps would be avoided by involving a supervised automatic pre-classification step, where manual classification would only be performed for a rather small number of ossicles based on several diagnostic properties (e.g., intensity/density, geometry, size), and then the manually-classified ossicles would act as training sets for semi-automatic classification of the remaining ossicles. We are planning this in future work.
– In our *Atrina* segmentation, knowledge of the biological system proved necessary to achieve a largely automated, high-quality segmentation, guiding “smart” merging of prisms in a physiologically-relevant orientation (i.e., the direction of growth). This approach demonstrates how, even in workflows that rely on manual segmentation to a very small degree, “expert knowledge” of a system can play an important role, through the incorporation of system-specific rules into the segmentation process or a downstream (semi-)manual proofreading step.

We hope that our presentation of this workflow will make modern segmentation approaches more visible and accessible to a wider audience, while encouraging interdisciplinary collaborations for visual data analysis problems in biological systems. We believe such cross-disciplinary interactions are the key for more effective visual data processing and, thereby, richer, more statistically-powerful data analyses.

## Notes

From the symposium “Adaptation and Evolution of Biological Materials” presented at the annual meeting of the Society for Integrative and Comparative Biology, January 3–7, 2019 at Tampa, Florida.
